# Exposure to outdoor cigarette advertisements and cigarette retailers near Indonesian schools: Density, proximity, and students’ self-report of exposure

**DOI:** 10.18332/tpc/194683

**Published:** 2024-11-21

**Authors:** Erni Astutik, Ηario Megatsari, Karin Gandeswari, Susy Katikana Sebayang, Siti Rahayu Nadhiroh, Santi Martini

**Affiliations:** 1 Department of Epidemiology, Biostatistics, Population Studies and Health Promotion, Faculty of Public Health, Universitas Airlangga, Surabaya, Indonesia; 2 Research Group for Health and Well-being of Women and Children, Universitas Airlangga, Surabaya, Indonesia; 3 Research Group Tobacco Control, Universitas Airlangga, Surabaya, Indonesia; 4 Faculty of Public Health, Universitas Airlangga, Surabaya, Indonesia; 5 Faculty of Health, Medicine and Life Sciences, Universitas Airlangga, Banyuwangi, Indonesia; 6 Department of Nutrition, Faculty of Public Health, Universitas Airlangga, Surabaya, Indonesia

**Keywords:** adolescent, behavior, tobacco, smoking, public health, addictive

## Abstract

**INTRODUCTION:**

Adolescents are vulnerable to tobacco advertising, promotion, and sponsorships (TAPS). The presence of TAPS, specifically outdoor cigarette advertisements (OCA) and cigarette retailers (CR), impacts adolescent smoking behavior. This study examined the presence of OCA and CR near Indonesian schools and students’ report of their TAPs exposure.

**METHODS:**

We conducted a cross-sectional study in the three diverse districts (Serang, Banyuwangi, and East Lombok) and a city district (Padang), Indonesia on September 2022–April 2023. In each district, we selected the three subdistricts with the most schools and the highest population density. Enumerators mapped all outdoor OCA and CR using Global Positioning System (GPS) devices. In selected schools within the study areas, we surveyed 6715 students about their TAPs exposure using face-to-face interviews. Students were selected using multistage cluster sampling. Data were analyzed descriptively using STATA 14.2.

**RESULTS:**

Of the 21460 retailers identified in the subdistricts, 30.4% were CR. The proportion of CR varied by district, between 24.8% and 40.7%. East Lombok had the highest percentage. Additionally, Banyuwangi had the highest density of CR. There were 13660 OCA points (district range 1918–6472). Around schools, banners were the most common OCA form (62.1%). Posters were second (32.8%). The retailers who sold the most cigarettes in the four districts were in kiosks. The density of CR and OCA per km^2^ increased as the distance from schools decreased. Students’ self-reported exposure to tobacco products was higher from OCA and CR than from the internet, television, and magazines/newspapers.

**CONCLUSIONS:**

OCA and CR are ubiquitous around schools. The Indonesian government should assess OCA and CR regulations and limit OCA and CR close to schools.

## INTRODUCTION

Globally, tobacco usage is the largest preventable cause of mortality and disability^1^. In 2010, the World Health Organization (WHO) estimated that tobacco use, primarily smoking, led to 4.9 million premature deaths per year^1^. This increased to 7.1 million in 2016. Assuming that present cigarette consumption rates remain stable, that figure will reach eight million by 2030^2^. The regions with the most smokers are South-East Asia Region (SEARO) and the Western Pacific, contributing about 6.4 million and 4.7 million smokers, respectively.

Indonesia is a major contributor to SEARO’s tobacco pandemic^3^. Within Indonesia, the smoking prevalence among youth aged 13–18 years is 38.3%. This is substantially higher than the rates seen in other SEARO countries, such as Malaysia (20.6%), Thailand (17.2%), and Myanmar (17%)^3^. Smoking prevalence among Indonesian youth aged 10–18 years grew from 7.2% in 2013 to 9.1% in 2018^4^.

Tobacco companies invest millions of dollars annually to market cigarettes through television, radio, cinema, newspapers, magazines, point of sale, outdoor displays, and the internet^5^. Indonesia is among the few ASEAN countries that have not completely prohibited tobacco advertising, promotion, and sponsorships (TAPS)^6^. Several studies have found a correlation between TAPS and youth smoking behavior^7,8^. Empirical evidence indicates that advertising and promotion by tobacco companies significantly affect adolescents to initiate tobacco use. Adolescents exposed to tobacco promotion frequently perceive the advertisements as attractive. Tobacco advertisements render smoking attractive, potentially heightening adolescents’ inclination to smoke^9^. WHO considers banning TAPS to be a necessary part of a global movement to overcome the tobacco epidemic^1^.

Studies conducted in Indonesia have documented outdoor cigarette advertisements (OCA) near public places, such as schools, health facilities, and religious sites^10-12^, the most recent was in 2020^8^. Significant correlations existed between teenage smoking prevalence and the density and proximity of outdoor tobacco advertising in Indonesia, although no such relationships were seen among adults. This underscores the necessity of implementing a ban on outdoor tobacco advertising, particularly in proximity to schools, in Indonesia and elsewhere. Other assessments of TAPS and cigarette retailers (CR) around schools in Indonesia are few^13^. This study provides a recent estimate of the current state of tobacco advertisements and retailers around selected schools in Indonesia. Specifically, it describes the density and proximity of OCA and CR near Indonesian schools and summarizes co-located students’ self-reported OCA exposure.

## METHODS

### Study design

Our study was cross-sectional, conducted between September 2022 and April 2023 in three selected districts (Serang, Banyuwangi, and East Lombok) and a city district (Padang). These districts all have some form of smoke-free area regulations. Serang and East Lombok have loose tobacco control policies. Padang and Banyuwangi have tight tobacco control policies. Specific local regulations ban tobacco advertisements^14^.

### Student population and sample

Our student survey sample was selected using multistage cluster sampling. First, within each of the four districts, we selected the three subdistricts with the highest number of schools and greatest population density. The selected subdistricts were Padang Barat, Padang Timur, and dan Koto Tangah (in Padang city); Cikande, Kramatwatu, and Kragilan (in Serang district); Muncar, Genteng, and Banyuwangi (in Banyuwangi district); and Aikmel, Pringgabaya, and Selong (in East Lombok district). Then, we identified all junior and high schools in these subdistricts. Next, we selected schools using proportional random sampling. The total number of schools in Padang, Serang, Banyuwangi, and East Lombok was 110, 108, 117, and 171, respectively. After selecting schools, we randomly selected two classes (each grade level at both junior and senior high school) in each school. All students in the selected classes were interviewed. Interviews were conducted in Bahasa. After missing data were excluded, 6715 students were interviewed.

### Cigarette retailers, outdoor cigarette advertisements, and survey data collection

We identified all cigarette retailers and outdoor cigarette advertisements in the study areas. Global Positioning System (GPS) devices were used to map schools, OCA, and CR locations, and Google Maps was used to identify and validate area boundaries. We recruited 37 local enumerators for the various districts and cities. A project researcher trained enumerators. Enumerators walked and rode motorcycles to collect the needed GPS data.

The enumerators also conducted the student surveys. Surveys were conducted in the schools, and parental permission was obtained before gathering survey data.

Data were collected using KoboToolBox^15^. Field coordinators and supervisors’ quality assured the data. The field coordinator checked the enumerators’ daily data collection count and reported to the supervisor. The supervisor verified the number of data entries in the database system. To ensure accuracy and validity, the quality control team reviewed a 10% random sample of the OCA and CR data collected by the enumerators, and reinterviewed 10% of each enumerator’s students.

### Variables

For the district OCA and CR survey, variables compiled included the total number of CR and OCA and their types. OCA types were posters, banners, billboards, powerwalls, stickers, store name boards, and others. CR types included stores, kiosks, street retailers, mini markets, supermarkets, and others.

We measured OCA and CR school proximity, as well as calculated OCA and CR density. We measured OCA proximity as the closest OCA to the school in meters. OCA density was the total number of OCA in km^2^ within three radii: 0–100 m, 0–300 m, and 0–500 m. CR proximity was measured as the closest CR to the school in meters. CR density was the total number of CR within the three radii in km^2^.

The student survey included five self-reported student exposures to tobacco products (Supplementary file). We assessed exposure to tobacco products based on four types of exposure, old media (television, newspaper, or magazine), new media (internet), OCA, and CR.

### Data analysis

Data were analyzed descriptively using STATA 14.2. Descriptive analysis was employed to examine the presence of OCA and CR near schools and students’ reports of their TAPs exposure.

## RESULTS

### Cigarette retailers

We identified 21460 retailers. Of these, 30.4% were CR. Across all locations, the proportion of CR varied between 24.8% and 40.7%. East Lombok had the highest proportion of CR (40.7%). Banyuwangi had the highest density, 9.96 per km^2^. Based on the number of children and adolescents, Banyuwangi also had the highest density at 2.6 per 1000 adolescents. Among the four study areas, kiosks were the most common type of retailer (Table 1).

**Table 1:** Distribution of the number of schools and cigarette retailers and outdoor cigarette advertisement characteristics, by district, September 2022 to April 2023, Indonesia

*Characteristics*	*Padang*		*Banyuwangi*		*Serang*		*East Lombok*	
	*n*	*%*	*n*	*%*	*n*	*%*	*n*	*%*
**Total number of schools**	110		117		108		171	
**Total population**	317812		346431		290990		192857	
**Area** (km^2^)	247.4		258.5		135.5		242.9	
**Cigarette retailers**								
Total number of retailers	2844		9598		5074		3944	
Total number of CR	1087	38.2	2574	26.8	1256	24.8	1606	40.7
CR per km^2^	4.4		9.96		9.3		6.6	
CR per 1000 children and adolescents	1.1		2.6		1.3		1.6	
Cigarette retailers by type	1087		2574		1256		1606	
Stores	209	19.2	525	20.4	373	29.7	161	10.0
Kiosks	742	68.3	1865	72.5	802	63.9	1229	76.5
Street retailers	81	7.5	52	2.0	1	0.1	73	4.6
Mini markets	24	2.2	52	2.0	75	6.0	63	3.9
Supermarkets	8	0.7	4	0.2	0	0.0	0	0.0
Other	23	2.1	76	3.0	5	0.4	80	5.0
**Outdoor cigarette advertisements**								
Total number of OCA	2014		6472		1918		3256	
OCA per km^2^	8.1		25.0		14.2		13.4	
OCA per 1000 children and adolescents	2.0		6.5		1.9		3.3	
**Outdoor cigarette advertisements by type**	2014		6472		1918		3256	
Poster	595	29.5	2457	38.0	230	7.1	1086	33.4
Banner	1264	62.8	3576	55.3	1544	47.4	1871	57.5
Billboard	1	0.1	24	0.4	64	2.0	31	1.0
Powerwall	15	0.7	33	0.5	5	0.2	4	0.1
Sticker	70	3.5	246	3.8	26	0.8	174	5.3
Store name board	8	0.4	31	0.5	37	1.1	61	1.9
Other	61	3.0	105	1.6	12	0.4	29	0.9

### Outdoor cigarette advertisements

OCA were observed at 13660 points (range per district: 1918–4472). Of the OCA types, banners were the most common around schools (62.1%). Posters were second (32.8%). Padang had the highest percentage of cigarette banners (62.8%.) Banyuwangi had the highest percentage of posters (38.0%) (Table 1).

In East Lombok, 60.2% of all schools had at least 1 cigarette retailer point within a 100 m radius. More than 90% of schools had at least one within a 0–300 m radius. This is only slightly better than in Banyuwangi, where 76.9% of schools had at least one within a 0–100 m radius, 98.4% of schools had at least one within a 0–300 m radius, and all schools had at least one within a 0–500 m radius. In Serang, the results were similar to Banyuwangi’s where 71.3% of schools had at least one within a 0–100 m radius, and more than 90% of schools had at least one within 0–300 m. Padang City was slightly better than the other three districts; half of the schools (51.8%) had at least 1 cigarette retailer point within a 0–100 m radius, 88.2% of schools had at least one within a 0–300 m radius, and 93.6% of schools had at least one within a 0–500 m radius (Table 2).

**Table 2:** Density of cigarette retailers and outdoor cigarette advertisements near schools by district, September 2022 to April 2023, Indonesia

*Variables*	*Number of schools*	*0–100 m*				*0–300 m*				*0–500 m*			
		*Schools with at least 1 CR or OCA*	*Percent with at least 1 CR or OCA*	*Mean number of CR or OCA*	*Mean density (per km^2^)*	*Schools with at least 1 CR or OCA*	*Percent with at least 1 CR or OCA*	*Mean number of CR or OCA*	*Mean Density (per km^2^)*	*Schools with at least 1 CR or OCA*	*Percent with at least 1 CR or OCA*	*Mean number of CR or OCA*	*Mean density (per km^2^)*
**Location of schools to cigarette retailers**													
**Overall**	506	327				463				483			
Padang	110	57	51.8	1.0	33.4	97	88.2	9.5	33.7	103	93.6	23.6	29.8
Banyuwangi	117	90	76.9	1.8	59.3	115	98.3	10.1	36.0	117	100.0	23.2	29.4
Serang	108	77	71.3	2.1	67.8	97	90	8.2	29.4	99	91.7	16.2	20.5
East Lombok	171	103	60.2	1.6	52.3	154	90.1	8.7	31.1	164	95.9	19.2	24.3
**Location of schools to outdoor cigarette advertisements**													
**Overall**	506					461				485			
Padang	110	45	40.9	1.5	48.1	97	88.2	13.9	49.5	106	96.4	37.2	47.1
Banyuwangi	117	83	70.9	4.3	137.6	114	97.4	22.1	78.9	117	100.0	49.1	62.1
Serang	108	77	71.3	2.8	88.7	97	89.8	11.4	40.8	98	90.7	22.6	28.7
East Lombok	171	108	63.2	3.0	97.5	153	89.5	17.9	63.9	164	95.9	40.0	50.6

CR: cigarette retailers. OCA: outdoor cigarette advertisements.

In all study locations, the density of CR/km^2^ increased as the distance from schools decreased. The density from the study schools ranged from 20.5–29.8 CR/km^2^ within 0–500 m, 29.4–36.0 within 0–300 m, and 33.4–67.8 within 0–100 m. The increase in CR/km^2^ was higher in Banyuwangi and Serang, than in Padang and East Lombok (Table 2).

The OCA density pattern was the same as the CR density pattern, i.e. as the distance from the schools decreased, the OCA density also increased. At a radius of 0–100 m, 71.3% of the schools in Padang, 70.9% in Banyuwangi, and 63.2% in East Lombok had at least 1 outdoor cigarette advertisement. Serang was the one exception, where only 40.9% of schools had one. Within a radius of 0–300 m, more than 88% of all schools in the study locations had at least 1 outdoor cigarette advertisement. Within 0–500 m, more than 90% of the schools had at least one, and every school in Banyuwangi had at least one. The density pattern differed somewhat in Padang, where at a radius of 0–100 m, the OCA/km^2^ slightly decreased compared to the density at a radius of 0–300 m. Within the 0–100 m radius, the highest OCA/km^2^ was in Banyuwangi (137.6), followed by East Lombok (97.5), Serang (88.7), and Padang (48.1) (Table 2).

Students were more exposed to tobacco products through the internet (range per district: 18.6–66.0%) than through television (range per district: 20.6–38.3%), magazines, or newspapers (range per district: 0.8–7.6%). However, exposure to tobacco products from the internet was lower than that from OCA (range per district: 75.2–85.7%) and CR (range per district: 80.2–95.5%). The highest tobacco exposure the students reported was OCA and CR (Table 3).

**Table 3:** Distribution of students’ self-reported tobacco product exposure, September 2022 to April 2023, Indonesia (N=6715)

*Variables*	*Responses*	*Padang*			*Banyuwangi*			*Serang*			*East Lombok*		
		*N=2095*			*N=1833*			*N=1376*			*N=1411*		
		*n*	*%*	*95% CI*	*n*	*%*	*95% CI*	*n*	*%*	*95% CI*	*n*	*%*	*95% CI*
**Old media**													
Over the last 30 days, did you see anyone smoking on TV shows or movies?	No	669	31.9	30.0–34.0	550	30.0	27.9–32.1	655	47.6	45.0–50.2	433	30.7	28.3–33.1
	Yes	792	37.8	35.8–39.9	465	25.4	23.4–27.4	283	20.6	18.5–22.8	541	38.3	35.8–40.9
	I did not watch television or films	634	30.3	28.3–32.3	818	44.6	42.4–46.9	438	31.8	29.4–34.3	437	31.0	28.6–33.4
Over the last 30 days, did you see any cigarette product advertisement in newspapers or magazines?	No	261	12.5	11.1–13.9	323	17.6	15.9–19.4	347	25.2	23.0–27.6	144	10.2	8.7–11.9
	Yes	159	7.6	6.5–8.8	86	4.7	3.8–5.8	11	0.8	0.4–1.4	44	3.1	2.3–4.2
	I did not see any newspapers or magazines	1675	80.0	78.2–81.6	1424	77.7	75.7–79.5	1018	74.0	71.6–76.2	1223	86.7	84.8–88.4
**New media**													
Over the last 30 days did you see any cigarette product advertisement on the internet?	No	665	31.7	29.8–33.8	887	48.4	46.1–50.7	881	64.0	61.5–66.5	760	53.9	51.3–56.5
	Yes	1381	66.0	63.9–67.9	860	46.9	44.6–49.2	256	18.6	16.6–20.8	479	34.0	31.5–36.5
	I did not access the internet	49	2.3	1.8–3.1	86	4.7	3.8–5.8	239	17.4	15.5–19.5	172	12.2	10.6–14.0
**Outdoor cigarette advertisements**													
Over the last 30 days, did you see any cigarette product advertisement on billboards?	No	249	11.9	10.6–13.3	341	18.6	16.9–20.5	303	22.0	19.9–24.3	167	11.8	10.3–13.6
	Yes	1795	85.7	84.1–87.1	1451	79.2	77.2–81.0	1035	75.2	72.9–77.4	1193	84.6	82.6–86.3
	I did not see any billboards	51	2.4	1.9–3.2	41	2.2	1.7–3.0	38	2.8	2.0–3.8	51	3.6	2.8–4.7
**Cigarette retailers**													
Over the last 30 days, did you see any cigarette products in stalls/kiosks/shops/etc.)?	No	226	10.8	9.5–12.1	169	9.2	8.0–10.6	262	19.0	17.1–21.2	159	11.3	9.7–13.0
	Yes	1850	88.3	86.9–89.6	1659	90.5	89.1–91.8	1103	80.2	78.0–82.2	1234	87.5	85.6–89.1
	I did not visit any stalls/kiosks/shops/etc.	19	0.9	0.6–1.4	5	0.3	0.1–0.7	11	0.8	0.4–1.4	18	1.3	0.8–2.0

## DISCUSSION

### General findings

Our study found that 30.4% of retailers were CR. The number of OCA observed was 13660 points. The density of CR and OCA per km^2^ increased as the distance from schools decreased. Students’ self-reported exposure to tobacco products showed that exposure from OCA and CR was higher than that from the internet, television, magazines, or newspapers. This means that the students were surrounded by various TAPS, which increases the possibility of initiating or maintaining smoking behavior. Tobacco advertising is a form of TAPS that is used by the cigarette industry to promote their product, enhance sales, and expand their youth market. They use both conventional and social media to do so. Therefore, if anti-TAPS policies do not include a comprehensive ban, the tobacco industry will find alternative means of promoting their products^16^.

### Comparison with other studies and possible mechanisms

Our study found that 30.4% of retailers were CR. In all four districts, kiosks were the most common type of retailer. This resembles results in Bali, Indonesia, where 77.8% of CR were kiosks^13^. CR play a crucial role in increased access to tobacco products^17^. The presence of CR around schools may prompt children and adolescents to smoke and increase their perception of the availability of tobacco products^13,18^. In Western Australia, a ban on the display of tobacco products reduced smoking prevalence^19^. Many people, especially children and youth, are exposed to the presence of cigarette products, which makes them interested in buying the products^20^. The presence of CR around educational institutions was found to lead to a higher risk of tobacco-related problems in Thai youth^21^.

In our study, the density of CR per km^2^ increased with decreasing distance from schools. We also found that 52–77% of the schools in all districts examined had at least 1 cigarette retailer point within a 100 m radius. Similar results were found in Bali^13^, Banyuwangi^22,23^, as well as Depok^24^. The proportion in our study was higher than that found in Thailand and elsewhere in Indonesia^25^. In Depok, West Java, Indonesia, a 40% higher density of CR was observed in areas near educational facilities^24^. In Ontario, Canada, smoking behavior was found to be significantly influenced by access to tobacco products. A major contributing factor to youth smoking is the high density of CR around schools^20^. The high density we found probably increases youth smoking prevalence in Indonesia^26^. The higher density of CR around schools indicates that CR were allowed to display tobacco products near schools and undoubtedly these displays are part of the tobacco industry’s programs to enhance the sales of their tobacco product specifically targeted at youth^27^,^28^.

The total number of OCA observed was 13660 and in any one district ranged between 1918 and 6472. This means that the students were surrounded by various TAPS. A previous study in Semarang found 3453 OCA points^29^. In our study, banners were the most common form of OCA around schools. In Banda Aceh, Indonesia, banners were also the most commonly found type of OCA^30^. However, in Surabaya, Indonesia, billboards were the most frequent type encountered OCA^11^. Surabaya is the capital of East Java Province and is the second-largest metropolitan city in Indonesia. This metropolitan environment may explain the dominance of billboards.

Similar to our results for CR, we found that as the distance from the schools decreased, OCA density increased. Likewise, in Semarang, Indonesia, OCA density was higher near schools^29^. In addition, a study conducted by Sebayang et al.^31^ in Surabaya and Banyuwangi, Indonesia, found that the density of outdoor tobacco advertising increased as the distance to an educational facility used by children and adolescents decreased. Research among Thai youth identified a high risk for tobacco-related problems due to their exposure to tobacco product advertising near educational institutions^25^.

According to students’ self-reported exposure to tobacco products, exposure from OCA and CR was higher than exposure through the internet, television, magazines, or newspapers. These results differ from a 2017 Indonesian study which identified high TAPS exposure from the Instagram (29.6%). Among offline types of exposure to TAPS, OCA (54.4%) was the second-highest exposure following television (74%)^8^. This difference might be because our research was conducted after the COVID-19 pandemic, between September 2022 and March 2023. During the pandemic, the government imposed restrictions on activity and travel^32,33^. Thus, children and adolescents, bored from being at home, tended to increase their outdoor activities, which more frequently exposed them to tobacco products through OCA and CR.

### Limitations

Our research does not represent all of Indonesia, due to differences in the geographical, social, and economic characteristics of the regions and because only four districts were studied. The sampling strategy used here involved selecting districts with high population densities and high school densities, which may contribute to a higher likelihood of OCA and CR in those areas. Additionally, the cross-sectional study design does not allow for the establishment of causal relationships between the variables studied. The use of self-reported information may introduce potential biases in this study. Further research is needed to examine broader and more representative areas in Indonesia.

### Policy implications

This study demonstrated that TAPS-related policies in Indonesia remain weak. The implementation of Presidential Regulation No. 109 of 2012, Article 39, and Law No. 32 of 2002, Article 46, concerning broadcasting, needs to be strengthened to eliminate cigarette advertising and promotions in communities, especially near children and adolescents. The government should regulate tobacco access, particularly for vulnerable groups, including children and adolescents. Restricting the proximity of CR and OCA to school premises can reduce the exposure to tobacco products among children and adolescents^25^, and thus reduce the prevalence of smoking among adolescents and children. To strengthen these efforts, comprehensive tobacco control measures, including a prohibition of outdoor tobacco advertising, are necessary^34,35^.

## CONCLUSIONS

In the surveyed areas in Indonesia, OCA and CR are ubiquitous around schools. Additionally, according to students’ self-reports, exposure to OCA and CR was greater than exposure from the internet, television, and magazines/newspapers.

## Supplementary Material



## Data Availability

Raw data that supports the findings of this study are available from the corresponding author, upon reasonable request.
