# Supplementary material for: Exposure to outdoor cigarette advertisements and cigarette retailers near Indonesian schools: Density, proximity, and students’ self-report of exposure
Source: Tob Prev Cessat. 2024 Nov 21;10:10.18332/tpc/194683. doi: 10.18332/tpc/194683 (PMC11580535; doi:10.18332/tpc/194683)
Supplement: Supplementary file 1 [file TPC-10-58-s1.pdf]

Supplementary File. Self-reported students' tobacco products exposure survey items

Survey Question	Responses
**Old Media**	
Over the last 30 days, did you see anyone smoking on TV shows or in movies?	No<br>Yes<br>I did not watch television or films over the last 30 days
Over the last 30 days, did you see any cigarette product advertisements in a newspaper or magazine?	No<br>Yes<br>I did not see a newspaper or magazine over the last 30 days
**New Media**	
Over the last 30 days, did you see any cigarette product advertisements on the internet?	No<br>Yes<br>I did not access the internet over the last 30 days
**Outdoor Cigarette Advertisements**	
Over the last 30 days, did you see any cigarette product advertisements on billboards?	No<br>Yes<br>I did not see billboards over the last 30 days
**Cigarette Retailers**	
Over the last 30 days, did you see any cigarette products in a stall/kiosk/shop/etc.)?	No<br>Yes<br>I did not visit a stall/kiosk/shop/etc. over the last 30 days

© 2024 Astutik E. et al.
